# The Longitudinal Impact of Daytime Dysfunction on Adolescent Depressive and Anxiety Symptoms: A Random Intercept Cross‐Lagged Panel Study

**DOI:** 10.1002/brb3.71303

**Published:** 2026-03-10

**Authors:** Na Zhang, Chenguang Ji, Shiyi Tang, Tiancheng Li, Chengcong Wu, Ziyue Zhu, Jiazhao Li, Yonghui Huang, Danyue Feng, Dongyan Ding, Wenjuan Wang

**Affiliations:** ^1^ Bengbu Medical University Bengbu Anhui China; ^2^ Fuyang Normal University Fuyan Anhui China

**Keywords:** daytime dysfunction, emotional distress, random‐intercept cross‐lagged panel model, social ostracism

## Abstract

**Introduction:**

Daytime dysfunction is a significant manifestation of sleep problems in adolescents, which increases individuals' negative emotional experiences by weakening their cognitive regulation and social adaptation. Meanwhile, emotional distress, such as depressive and anxiety symptoms, may further exacerbate daytime dysfunction, creating a negative cycle. Therefore, the present study employed a longitudinal follow‐up design to explore the bidirectional relationship between daytime dysfunction and depressive and anxiety symptoms in adolescents.

**Methods:**

This three‐wave longitudinal study used stratified random sampling to recruit 936 adolescents (50.96% girls; mean age = 14.37 ± 1.32 years) from four secondary schools in Bengbu, Anhui Province, China. Data were collected between September 2023 and September 2024 at 4‐month intervals. Standardized questionnaires measured daytime dysfunction, social ostracism, depressive symptoms, and anxiety symptoms. Random intercept cross‐lagged panel models (RI‐CLPM) were used to examine within‐ and between‐person associations and to test the longitudinal mediating roles of two distinct forms of social ostracism.

**Results:**

At the between‐individual level, the results indicated a significant positive association between daytime dysfunction and depressive and anxiety symptoms (*r* = 0.319–0.495, *p* < 0.001), and a significant positive association between social ostracism and depressive and anxiety symptoms (*r* = 0.120–0.504, *p* < 0.05). The link between social ostracism and daytime dysfunction was weaker, mainly involving social rejection. At the within‐individual level, social neglect at T2 significantly mediated the longitudinal relationship between daytime dysfunction at T1 and anxiety symptoms at T3.

**Conclusions:**

These findings suggest the negative impact of daytime dysfunction on adolescents' depressive and anxiety symptoms and highlight the role of social neglect in social ostracism on this influence.

## Graphical abstract

Longitudinal analyses using a random intercept cross‐lagged panel model (RI‐CLPM) indicate that daytime dysfunction predicts later increases in adolescents'  depressive and anxiety symptoms, mediated by increases in social ostracism.

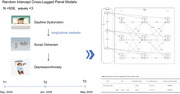

## 1 Introduction

### 1.1 The Prevalence and Impact of Adolescent Daytime Dysfunction

Sleep disturbances are a major public‐health concern among adolescents, encompassing irregular sleep–wake patterns, insufficient duration, difficulty initiating sleep, daytime sleepiness, and nightmares (Gariepy et al. 2020). Prevalence estimates range from 30% to 70% (Patte et al. 2017), and recent evidence shows a persistent decline in sleep duration in youth populations (Chen et al. 2024; Poirier et al. 2024). In China, this trend is particularly severe due to academic pressure and late‐night study routines. According to the *China Sleep Research Report 2024*, the average sleep duration of high school students is 6.5 h, with many going to bed after 11:00 p.m. (Zhang et al. 2024). From a neurocognitive perspective, the dual‐process model (Borbély et al. 2016) suggests that increased sleep pressure impairs prefrontal executive functions (Diamond 2013), and insufficient sleep further induces daytime dysfunction, including attentional deficits, memory impairment, and reduced decision‐making capacity (Curtis et al. 2018).

According to the Pittsburgh Sleep Quality Index (PSQI), daytime dysfunction refers to symptoms such as fatigue, inattention, memory impairment, and emotional instability—all of which significantly interfere with daily functioning and task performance (Huang et al. 2024). With the rising prevalence of sleep problems among adolescents, daytime dysfunction has become a central research focus. Adolescents exposed to academic pressure, social anxiety, or emotional difficulties frequently exhibit varying degrees of functional impairment, which not only hinders academic performance but also has long‐term negative effects on mental health (Linkas et al. 2022).

Growing evidence indicates that persistent daytime dysfunction undermines cognitive capacities, weakens social adaptation, and further aggravates emotional distress (Wang et al. 2024). However, the mechanisms by which sleep‐related daytime dysfunction progresses to emotional sequelae over time remain poorly elucidated. Notably, extant research has predominantly characterized cross‐sectional or inter‐individual correlations, leaving the temporal dynamics and mechanistic pathways linking daytime dysfunction to anxiety and depressive symptoms insufficiently specified.

### 1.2 The Relationship Between Daytime Dysfunction, Depressive, and Anxiety Symptoms

In recent years, elevated levels of anxiety and depressive symptoms have been increasingly reported among adolescents worldwide. Large‐scale evidence indicates that anxiety and depression have shown a marked increase in youth populations over the past decade (Mcgorry et al. 2024), raising widespread concern about adolescent emotional health, particularly the role of sleep in emotional regulation. Insufficient sleep has been identified as a direct catalyst for emotional disturbances and mood disorders (Triantafillou et al. 2019). This association is supported by studies demonstrating the critical roles of rapid eye movement and slow‐wave sleep in affective modulation (Finan et al. 2017). The adverse impact of sleep deprivation is especially pronounced in adolescents, who are highly sensitive to disruptions in emotional stability (Tempesta et al. 2018). Moreover, a recent meta‐analysis further demonstrated that insufficient sleep intensifies emotional instability, particularly anxiety and depressive symptoms (Palmer et al. 2024).

There is a bidirectional relationship between insufficient sleep and emotional distress. For instance, inadequate sleep can heighten anxiety symptoms by increasing activity in neural regions such as the amygdala and insula, while anxiety symptoms can in turn disrupt sleep, creating a vicious cycle (Ball et al. 2013). Daytime dysfunction induced by poor sleep, including attentional deficits and reduced executive function, further compounds these difficulties by hindering adolescents’ ability to cope with academic and daily demands, thereby amplifying anxiety and depressive symptoms.

From a behavioral and cognitive perspective, daytime dysfunction resulting from insufficient sleep impairs attention, processing speed, and responsiveness, which may partly compromise adolescents' capacity to regulate emotional responses. When these deficits manifest in social contexts, they heighten vulnerability to adverse social encounters, which in turn amplify anxiety and depressive symptoms over time. This framework highlights that daytime dysfunction operates beyond neurocognitive sequelae to compromise social functioning, providing a coherent framework for understanding how poor sleep contributes to adolescent emotional problems.

### 1.3 The Mediating Role of Social Ostracism

Social ostracism refers to the experience of being excluded or rejected by others, thereby threatening one's need for belonging and connection (Lincoln et al. 2021). It is common in adolescent life, manifesting in peer exclusion, familial neglect, or social isolation. Research has shown that social ostracism undermines self‐esteem, self‐control, and self‐efficacy (Rajchert et al. 2024), all of which are closely associated with anxiety and depressive symptoms during adolescence.

Daytime dysfunction appears to heighten adolescents’ vulnerability to social ostracism. Impairments in attention, emotional reactivity, and behavioral responsiveness make it more difficult for adolescents to engage effectively in social interactions, increasing the likelihood of being excluded or overlooked. These negative social experiences, in turn, exacerbate emotional difficulties, providing proximal triggers for anxiety and depressive symptoms. Through this mechanism, daytime dysfunction may not only directly affect emotional states but also indirectly amplify anxiety and depressive symptoms via increased social vulnerability.

Two main forms of social ostracism are commonly identified: social neglect and social rejection (Li et al. 2017). Social neglect involves passive disregard or coldness, while rejection entails active exclusion or hostility (Gilman et al. 2013). Although both forms threaten basic psychological needs, they operate through distinct psychological and behavioral mechanisms (Zhang et al. 2018). According to Williams's temporal need‐threat model (Williams 2009), ostracism compromises four core needs—belonging, self‐esteem, control, and meaningful existence. When these needs are threatened, individuals experience intense affective reactions, such as anxiety and depressive symptoms. Social neglect primarily undermines belonging and existence, leading to social withdrawal and heightened depressive and anxious symptoms (Williams and Nida 2011), whereas social rejection directly damages self‐worth and perceived control through negative evaluation and cognitive distortions, thereby triggering anxiety symptoms (Downey and Feldman 1996).

From a neurobiological perspective, social exclusion exacerbates the risk of emotion regulation deficits and affective disorders by inducing structural and functional brain alterations, such as reduced hippocampal volume (Látalová et al. 2023). Converging evidence also indicates that chronic social ostracism promotes emotional dysregulation via neuroplastic alterations in frontolimbic circuitry. Specifically, social neglect has been associated with heightened sensitivity in the dorsal anterior cingulate cortex (dACC) and increased reactivity in the amygdala, both of which are closely linked to anxiety symptoms (Dewall et al. 2012; Roth et al. 2018). Rejection, by contrast, dampens ventral striatum activity and reward sensitivity, potentially increasing vulnerability to depressive symptoms (Quarmley et al. 2019). Thus, understanding these differential effects provides a basis for clarifying the mediating role of social ostracism in the impact of daytime dysfunction on emotional health and for distinguishing mechanisms underlying anxiety and depressive symptoms.

The social information processing (SIP) model provides a theoretical framework for understanding this mechanism (Dodge and Crick 1990). When cognitive and executive functions are impaired due to daytime dysfunction, the encoding, interpretation, attribution, and response selection of social cues are more prone to biases, increasing the risk of negative social experiences and subsequent emotional consequences. For example, sleep deprivation may impair prefrontal cortical function, reducing the ability to accurately perceive others’ emotions and intentions, leading adolescents to misinterpret neutral social signals as threatening. Such biases can trigger defensive or avoidant behaviors, increasing the likelihood of ostracism (van der Helm et al. 2010; Tashjian and Galván 2020; Holding et al. 2019).

Overall, this study aims to comprehensively examine the dynamic associations among daytime dysfunction, social ostracism, and emotional problems in adolescents. Using a random‐intercept cross‐lagged panel model (RI‐CLPM), it seeks to uncover the temporal pathways connecting these variables. In contrast to traditional cross‐sectional designs, the longitudinal framework captures within‐person changes across time, thereby addressing gaps in the existing literature and providing new empirical evidence for understanding the mechanisms of adolescent mental health.

### 1.4 Current Research

The present study aimed to investigate the longitudinal relationship between daytime dysfunction and emotional problems in adolescents, specifically depressive and anxiety symptoms, and to explore the underlying mechanisms. In particular, we examined whether social ostracism mediates the relationship between daytime dysfunction and emotional distress over time. Based on previous research and theoretical frameworks, we proposed the following hypotheses:

Hypothesis 1: At the between‐individual level of the RI‐CLPM, daytime dysfunction and social ostracism are each associated with depressive and anxiety symptoms, whereas daytime dysfunction is positively associated with social ostracism.
Hypothesis 2: At the within‐individual level of the RI‐CLPM, daytime dysfunction positively predicts depressive and anxiety symptoms, with social ostracism serving as a longitudinal mediator in this relationship.

## 2 Method

### 2.1 Participants

We conducted a three‐wave longitudinal study with stratified random sampling in four secondary schools (two junior‐high and two senior‐high) in Bengbu, Anhui Province, China. The primary aim was to examine the longitudinal relationships among daytime dysfunction, social ostracism, and depressive and anxiety symptoms in adolescents, with special attention to the potential mediating role of social ostracism.

**FIGURE 1:**
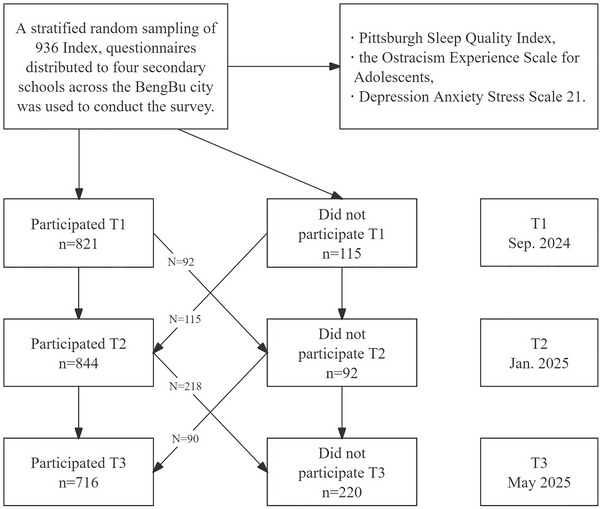
Participants flow through the study. T1 = Time 1, T2 = Time 2, T3 = Time 3.

The first wave of data collection (T1) occurred in September 2023, followed by two additional waves at 4‐month intervals (T2 and T3). At T1, 821 students completed the survey (attrition rate = 12.29%). At T2, 844 valid responses were obtained (attrition rate = 9.83%). At T3, 716 participants remained (attrition rate = 23.50%). In total, 936 adolescents were included in the final sample. Of these, 459 were boys (49.04%) and 477 were girls (50.96%), ranging in age from 11 to 17 years (M = 14.37). The sample included 475 urban students (50.75%) and 461 rural students (49.25%), as well as 102 boarding students (10.90%) and 834 non‐boarding students (89.10%). The participant selection flowchart is shown in Figure 1.

### 2.2 Measurements

#### 2.2.1 General Information Questionnaire

Participants reported their gender, residential location, grade level, and boarding status. These data were used to characterize the sample and were included as covariates in the analyses where appropriate.

#### 2.2.2 Pittsburgh Sleep Quality Index

Daytime dysfunction was assessed using the daytime components of the Pittsburgh Sleep Quality Index (PSQI) (Liu and Tang 1996). Two relevant to daytime functioning were extracted and rated on a 4‐point Likert scale, with higher scores indicating greater impairment. Cronbach's alpha values across T1, T2, and T3 were 0.795, 0.765, and 0.763, respectively, indicating acceptable internal consistency.

#### 2.2.3 Ostracism Experience Scale for Adolescents

Social ostracism was measured using the revised ostracism experience scale for adolescents (OES‐A) developed by Zhang et al. 2018. The scale comprises 11 items across two subscales: social neglect (five items) and social rejection (six items), rated on a 5‐point Likert scale (1 = “never” to 5 = “always”). Higher total scores indicate greater perceived ostracism. Cronbach's alpha coefficients at T1, T2, and T3 ranged from 0.742 to 0.893, demonstrating good reliability.

#### 2.2.4 Depression Anxiety Stress Scale‐21

This investigation utilized the Simplified Chinese adaptation of the Depression Anxiety Stress Scale‐21 (DASS‐21), originally developed by Lovibond and Lovibond 2002 and subsequently revised by Gong et al. 2010. The DASS‐21 is a self‐report instrument designed to assess the severity of emotional symptoms rather than to establish clinical diagnoses. Only the depression and anxiety subscales (seven items each) were used. Items were rated on a 4‐point Likert scale (0 = “did not apply to me” to 3 = “applied to me most of the time”), and raw scores were doubled. Clinical cutoffs were depression (10, 14, 21 for mild, moderate, and severe) and anxiety (8, 10, 15). Cronbach's alpha coefficients for depression and anxiety were 0.928 (T1), 0.942 (T2), and 0.958 (T3), indicating excellent internal consistency.

### 2.3 Procedure

The Human Research Ethics Committee of Bengbu Medical University approved the study (Ref. [2023] No. 661). Written informed consent was obtained from each adolescent and from at least one parent or legal guardian before data collection began.

Each wave of data collection took place in a quiet, school‐based computer classroom. Participants accessed the questionnaires through the secure online platform Wenjuanxing (www.wjx.cn). A trained researcher introduced the session, remained present throughout, and provided assistance as needed to ensure standardized administration and reliable responses.

### 2.4 Statistical Analysis

First, confirmatory factor analyses (CFA) were conducted to examine the longitudinal measurement invariance of each scale across the three time points. Little's MCAR test indicated that the data were missing completely at random, *χ^2^* = 26.632, *df* = 35, *p* = 0.844. Missing data were handled using full information maximum likelihood (FIML) estimation as implemented in Mplus.

Second, descriptive statistics and Pearson correlation analyses for key variables, including daytime dysfunction, social ostracism, depressive symptoms, and anxiety symptoms, were conducted using SPSS 26.0. In addition, repeated measures ANOVA was employed to examine changes in these variables across the three time points.

Third, intraclass correlation coefficients (ICCs) were calculated to determine the proportion of between‐person variance (Nie et al. 2019). The ICCs for daytime dysfunction, social neglect, social rejection, depressive symptoms, and anxiety symptoms were 51.2%, 41.7%, 47.0%, 57.3%, and 57.0%, respectively. As values exceeded 10%, the use of RI‐CLPM was justified (Yang et al. 2022).

Fourth, a longitudinal measurement equivalency was assessed in Mplus 8.3 to ensure consistency of the measurement instruments over time. In conducting the confirmatory factor analyses, we systematically tested three levels of measurement invariance: Configural Invariance, Metric Invariance, and Intercept Invariance. The overall model fit was assessed using four indices: CFI, TLI, RMSEA, and SRMR. Adopting the standards proposed by Hooper et al. 2007, a structural equation model was considered to meet the fit criteria when the CFI and TLI were above 0.90, the RMSEA was below 0.08, and the SRMR was below 0.08. When these thresholds were not met, a stepwise adjustment method (Ng et al. 2019) was applied to relax constraints on certain items, allowing the construction of a partial measurement equivalency model (Byrne et al. 1989).

**FIGURE 2:**
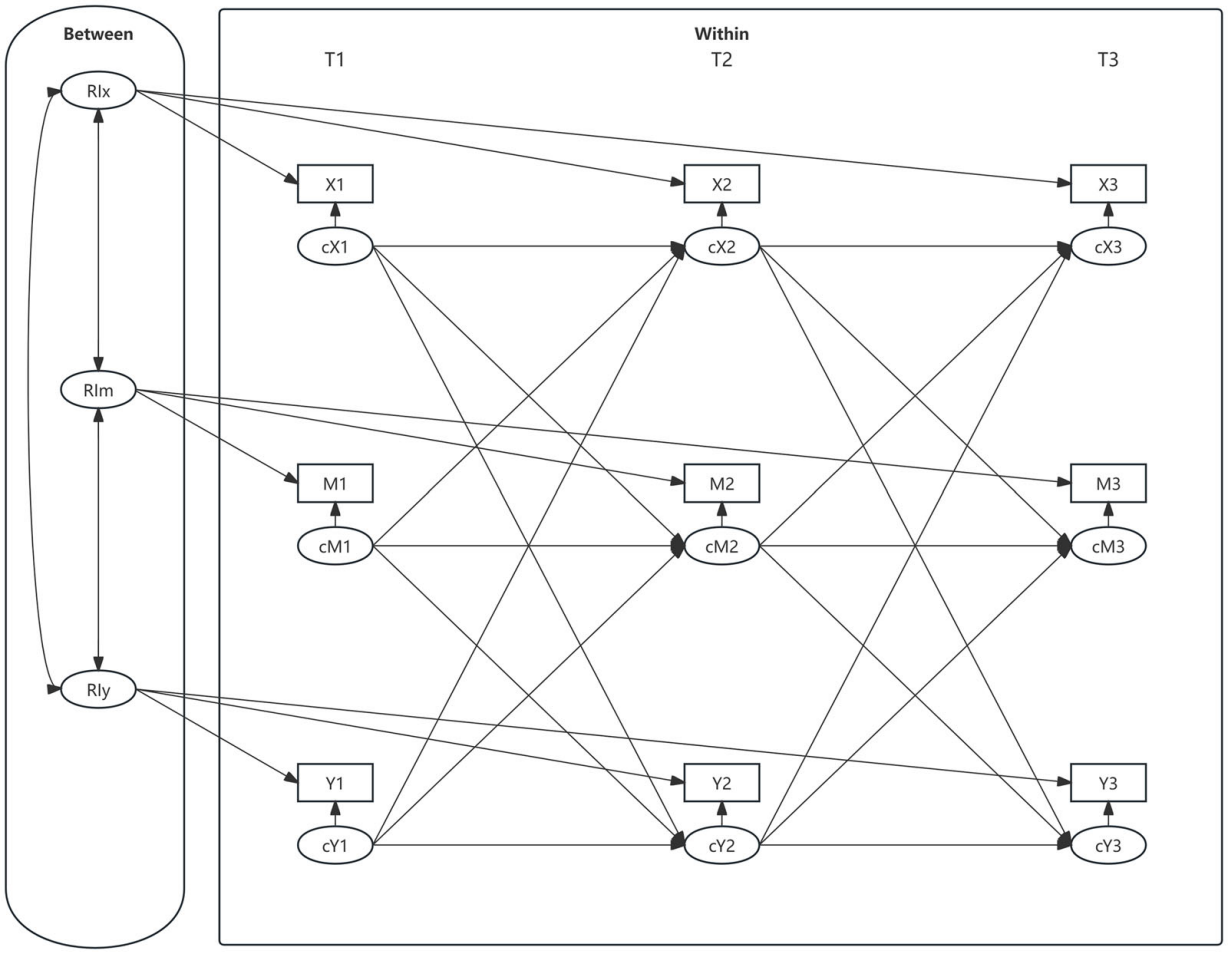
Diagram of a random intercept cross‐lagged model with mediation. X1–X3 represent the observed values of the independent variables from T1 to T3. M1–M3 represent the observed values of the intermediate variables from T1 to T3. Y1–Y3 represent the observed values of the dependent variables from T1 to T3. cX1–cX3 represent the slope of the within‐individual level independent variable from T1 to T3. cM1–cM3 represent the slope of the within‐individual level mediator variables from T1 to T3. cY1–cY3 represent the slope of the dependent variables at the within‐individual level from T1 to T3. RI—X, RI—M, and RI—Y represent the random intercept of the independent variables, mediator variables, and dependent variables, respectively. All variables at each time point represent observed composite scale scores rather than latent constructs; item parceling was not applied.

Finally, after establishing measurement equivalency, four RI‐CLPMs were constructed based on the research aims and hypotheses. All analyses were conducted using observed composite scores rather than latent variables, and item parceling was not applied. In these models, daytime dysfunction served as the independent variable, social neglect and rejection were modeled as mediators, and depressive and anxiety symptoms as outcome variables. Gender and grade level were controlled as covariates in all models. All RI‐CLPMs were estimated using the robust maximum likelihood estimator (MLR). As illustrated in Figure 2, the random intercept (RI) latent variable was constructed by extracting the stable components of each construct across all waves, representing individuals’ average levels and capturing stable between‐person differences‐analogous to trait‐level effects. After extracting the random intercept, the remaining residual components (c) reflect individual deviations from their average levels at each time point, capturing within‐person fluctuations comparable to state‐level effects (Hoffman and Stawski 2009; Orth et al. 2021). The model's fit was assessed by employing standardized fit indices, including CFI, TLI, and RMSEA, along with their corresponding fit statistics.

## 3 Results

### 3.1 Common Method Bias Test

Harman's single‐factor method was employed to examine the presence of common method bias across the three waves (Podsakoff et al. 2003). At Time 1 (T1), six factors had eigenvalues greater than 1, and the first unrotated factor accounted for 31.23% of the total variance. At Time 2 (T2), six factors emerged, with the first factor explaining 32.27% of the variance. At Time 3 (T3), five factors had eigenvalues exceeding 1, with the first factor accounting for 37.17% of the variance. As multiple factors emerged at each time point and none of the first factors explained more than 40% of the variance, the results suggest that common method bias was not a major concern.

### 3.2 Longitudinal Measurement Equivalency Testing

Measurement invariance across time was tested using four increasingly constrained models: configural, metric, intercept, and strict invariance. Following Chen's recommendations (2007), changes in CFI < 0.01 and RMSEA < 0.015 between nested models indicate acceptable invariance (Chen et al. 2024; Bentler 2006; Chen 2007). The results supported scalar invariance for all instruments, confirming that both factor loadings and intercepts remained stable over time, thus justifying longitudinal comparisons of latent means.

### 3.3 Main Analyses

Repeated measures ANOVA was conducted to examine whether there were significant changes across three waves in daytime dysfunction, perceived neglect, perceived rejection, anxiety symptoms, and depressive symptoms. The results are presented in Table 1.

**TABLE 1:** Mean and standard deviation of each variable, repeated measure ANOVA and multiple comparison results.

Variables	Times	M ± SD		*F* test		Multiple comparisons
			*F*	*p*	*η^2^*	
	T1	3.18 ± 1.859				
Daytime dysfunction	T2	3.59 ± 1.735	32.593	< 0.001	0.065	T2 > T1
	T3	3.63 ± 1.782				T3 > T1
	T1	21.96 ± 4.107				
Social rejection	T2	21.22 ± 4.400	20.246	< 0.001	0.042	T1 > T2
	T3	21.00 ± 4.861				T1 > T3
	T1	8.48 ± 3.358				
Social neglect	T2	9.52 ± 3.923	49.055	< 0.001	0.095	T2 > T1
	T3	9.62 ± 4.139				T3 > T1
	T1	12.50 ± 4.014				
Anxiety symptoms	T2	12.22 ± 4.272	9.071	< 0.001	0.019	T1 > T2 > T3
	T3	11.31 ± 4.457				
	T1	11.56 ± 4.045				
Depressive symptoms	T2	11.49 ± 4.157	3.162	< 0.05	0.007	T1 > T3
	T3	11.23 ± 4.414				T2 > T3

*Note*: T1 = Time 1, T2 = Time 2, T3 = Time 3.

All five variables showed significant main effects of time. Post hoc comparisons indicated that daytime dysfunction at T2 and T3 was significantly higher than at T1 (*F* (2,2381) = 32.593, *p* < 0.001, *η^2^* = 0.065). Social rejection was significantly higher at T1 than at T2 and T3 (*F*(2,2381) = 20.246, *p* < 0.001, *η^2^* = 0.042), while social neglect at T2 and T3 was significantly higher than at T1 (*F*(2,2381) = 49.055, *p* < 0.001, *η^2^* = 0.095). Anxiety symptoms were significantly higher at T1 than at T2 and T3 (*F*(2,2381) = 9.071, *p* < 0.001, *η^2^* = 0.019), and depressive symptoms were also significantly higher at T1 than at T2 and T3 (*F*(2,2381) = 3.162, *p* < 0.05, *η^2^* = 0.007).

Table 2 presents the correlation matrix of the main study variables. The results indicate that daytime dysfunction, anxiety symptoms, and depressive symptoms were moderately to strongly positively correlated across different waves (*r* = 0.319–0.495, *p* < 0.001), suggesting good longitudinal stability. Both social neglect and social rejection were significantly positively correlated with anxiety and depressive symptoms. at each time point (*r* = 0.120–0.504, *p* < 0.05). In contrast, their correlations with daytime dysfunction were relatively weaker, particularly for social rejection. Overall, the significant associations among these variables provide a foundation for subsequent path analyses.

**TABLE 2:** Correlation coefficient matrix of each variable.

	1	2	3	4	5	6	7	8	9	10	11	12	13	14
1.T1 daytime dysfunction	1													
2.T2 daytime dysfunction	0.574^***^	1												
3.T3 daytime dysfunction	0.499^***^	0.626^***^	1											
4.T1 social rejection	0.142^**^	0.058	‐ 0.021	1										
5.T2 social rejection	0.88	0.76	0.58	0.501^***^	1									
6.T3 social rejection	0.094^*^	0.092	0.144^**^	0.403^***^	0.567^***^	1								
7.T1 social neglect	0.264^***^	0.240^***^	0.170^***^	0.402^***^	0.245^***^	0.202^***^	1							
8.T2 social neglect	0.113^*^	0.158^***^	0.163^***^	0.254^***^	0.227^***^	0.226^***^	0.476^***^	1						
9.T3 social neglect	0.134^**^	0.066	0.150^**^	0.210^***^	0.156^**^	0.014	0.373^***^	0.497^***^	1					
10.T1 anxiety symptoms	0.495^***^	0.404^***^	0.405^***^	0.218^***^	0.116^*^	0.144^**^	0.443^***^	0.288^***^	0.246^***^	1				
11.T2 anxiety symptoms	0.383^***^	0.417^***^	0.427^***^	0.158^**^	0.190^***^	0.124^**^	0.404^***^	0.265^***^	0.345^***^	0.614^***^	1			
12.T3 anxiety symptoms	0.353^***^	0.389^***^	0.463^***^	0.126^**^	0.179^***^	0.164^***^	0.319^***^	0.240^***^	0.298^***^	0.484^***^	0.704^***^	1		
13.T1 depressive symptoms	0.456^***^	0.369^***^	0.348^***^	0.290^***^	0.204^***^	0.167^***^	0.504^***^	0.325^***^	0.278^***^	0.759^***^	0.528^***^	0.442^***^	1	
14.T2 depressive symptoms	0.404^***^	0.409^***^	0.411^***^	0.208^***^	0.200^***^	0.184^***^	0.424^***^	0.298^***^	0.310^***^	0.611^***^	0.789^***^	0.646^***^	0.668^***^	1
15.T3 depressive symptoms	0.319^***^	0.362^***^	0.451^***^	0.120^*^	0.188^***^	0.180^***^	0.277^***^	0.283^***^	0.298^***^	0.462^***^	0.617^***^	0.856^***^	0.513^***^	0.692^***^

*p < 0.05.

**p < 0.01.

***p < 0.001.

### 3.4 Random Intercept Cross‐Lagged Panel Model

#### 3.4.1 RI‐CLPM With Social Neglect as a Mediating Variable

**FIGURE 3:**
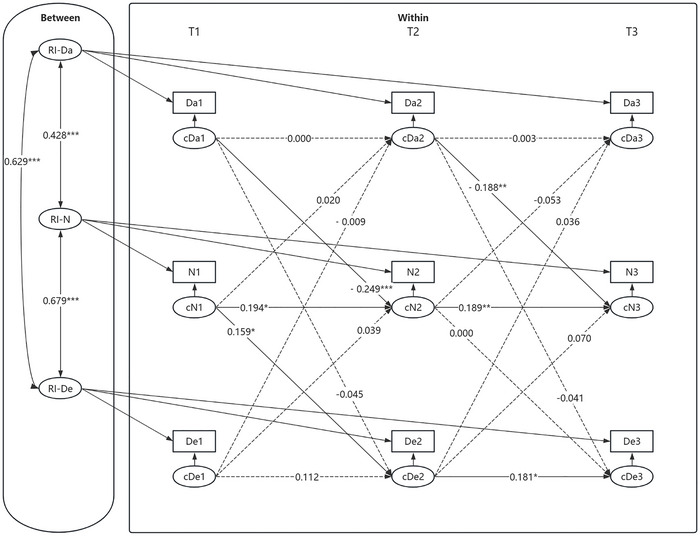
RI‐CLPM of daytime dysfunction, social neglect, and depressive symptoms. *p < 0.05, **p < 0.01, ***p < 0.001. Da1–Da3, N1–N3, and De1–De3 represent the observed values of daytime dysfunction, social neglect, and depressive symptoms from T1 to T3, respectively. cDa1–cDa3, cN1–cN3, and cDe1–cDe3 represent the within—person slopes of daytime dysfunction, social neglect, and depressive symptoms from T1 to T3. RI—Da, RI—N, and RI—De represent the random intercepts of daytime dysfunction, social neglect, and depressive symptoms, respectively. All variables at each time point represent observed composite scale scores rather than latent constructs; item parceling was not applied.

First, an RI‐CLPM was established to investigate the mediating role of social neglect between daytime dysfunction and depressive symptoms. The modeled relationships among variables are depicted in Figure 3. The model demonstrated good fit: *χ^2^/df* = 0.069*, RMSEA* [90% CI] = 0.000 [0.000, 0.000], CFI = 1.000, TLI = 1.017, SRMR = 0.001.

At the between‐individual level, daytime dysfunction was significantly associated with social neglect *(β* = 0.428, SE = 0.082*, p* < 0.001), indicating that adolescents with higher levels of daytime dysfunction were more likely to experience neglect. In turn, social neglect was positively associated with depressive symptoms *(β* = 0.679, SE = 0.067*, p* < 0.001), suggesting that adolescents who felt more neglected tended to report more depressive symptoms. A direct association was also found between daytime dysfunction and depressive symptoms *(β* = 0.629, SE = 0.051*, p* < 0.001).

At the within‐individual level, social neglect at T(n) significantly predicted social neglect at T(n+1), and depressive symptoms at T2 significantly predicted depressive symptoms at T3. For cross‐lagged effects, daytime dysfunction at T(n) significantly predicted social neglect at T(n+1). Additionally, social neglect at T1 significantly predicted depressive symptoms at T2. In contrast, the autoregressive paths for daytime dysfunction across all three time points were non‐significant, and the autoregressive coefficient for depressive symptoms was not significant from T1 to T2. Furthermore, other cross‐lagged paths did not reach statistical significance. The results of the mediating effect analysis showed that the mediating effects were not significant.

**FIGURE 4:**
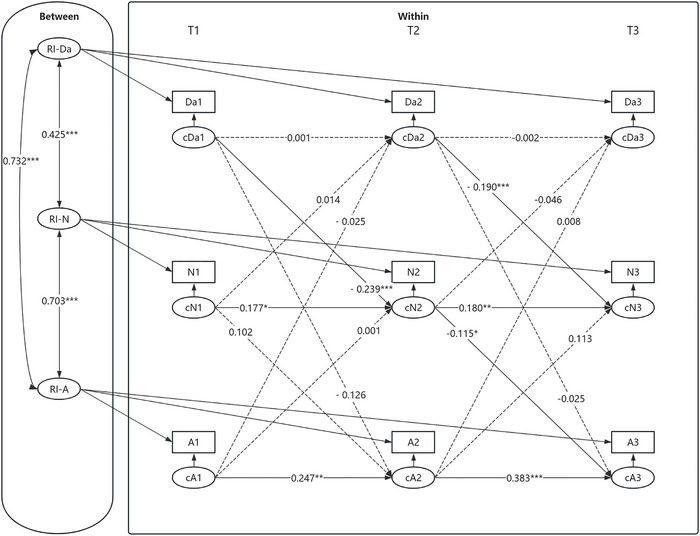
RI‐CLPM of daytime dysfunction, social neglect, and anxiety symptoms. *p < 0.05, **p < 0.01, ***p < 0.001. Da1–Da3, N1–N3, and A1–A3 represent the observed values of daytime dysfunction, social neglect, and anxiety symptoms from T1 to T3, respectively. cDa1–cDa3, cN1–cN3, and cA1–cA3 represent the within‐individual slopes of daytime dysfunction, social neglect, and anxiety symptoms from T1 to T3, respectively. RI—Da, RI—N, and RI—A represent the random intercepts of daytime dysfunction, social neglect, and anxiety symptoms, respectively. All variables at each time point represent observed composite scale scores rather than latent constructs; item parceling was not applied.

Second, an RI‐CLPM was established to investigate the mediating role of social neglect between daytime dysfunction and anxiety symptoms. The modeled relationships among variables are depicted in Figure 4. The model fit indices indicated a good fit: *χ^2^/df* = 2.873, RMSEA [90% CI] = 0.000 [0.000, 0.054], CFI = 1.000, TLI = 1.001, SRMR = 0.008.

At the between‐individual level, daytime dysfunction was significantly positively associated with social neglect (*β* = 0.425, SE = 0.078*, p* < 0.001). Moreover, social neglect was significantly positively associated with anxiety symptoms (*β* = 0.703, SE = 0.085*, p* < 0.001), indicating that adolescents who experienced higher levels of neglect reported more severe anxiety symptoms. Additionally, daytime dysfunction was strongly associated with anxiety symptoms (*β* = 0.732, SE = 0.071, *p* < 0.001), suggesting that higher levels of daytime dysfunction were related to more severe anxiety symptoms among adolescents.

At the within‐individual level, the autoregressive paths of social neglect and anxiety symptoms were significant across all three time points. In terms of cross‐lagged effects, daytime dysfunction at T(*n*) significantly predicted social neglect at T(*n*+1); additionally, social neglect at T2 significantly predicted anxiety symptoms at T3. In contrast, the autoregressive paths of daytime dysfunction were not significant at any time point. Furthermore, other cross‐lagged paths did not reach statistical significance. The mediating effect analysis revealed that the level of daytime dysfunction at T1 impacted anxiety symptoms at T3 through its influence on social neglect at T2. The results of the mediating effect tests are shown in Table 3.

**TABLE 3:** Bootstrap tests for the significance of the mediation effect.

Effect	Path	Standardized estimate	95% confidence interval	Proportion of mediating effects
Total mediation effect		0.025	0.007‐0.055	100.00%
	cDa1 → cN2 → cA3	0.025	0.007‐0.055	100.00%

*Note*: cDa1 represents the slope of daytime dysfunction from T1 to T3 at the within‐individual level. cN2 represents the slope of social neglect from T1 to T3 at the within‐individual level. cA3 represents the slope of anxiety symptoms from T1 to T3 at the within‐individual level.

#### 3.4.2 RI‐CLPM With Social Rejection as a Mediating Variable

**FIGURE 5:**
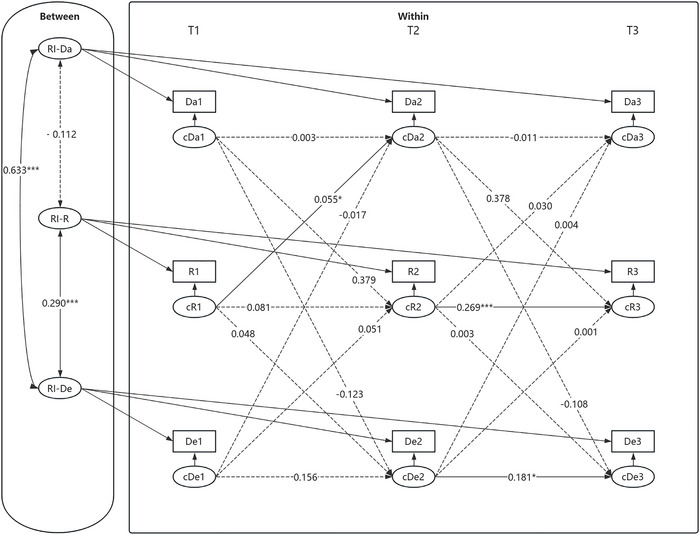
RI‐CLPM of daytime dysfunction, social rejection, and depressive symptoms. *p < 0.05, **p < 0.01, ***p < 0.001. Da1–Da3, R1–R3, and De1–De3 represent the observed values of daytime dysfunction, social rejection, and depressive symptoms from T1 to T3, respectively. cDa1–cDa3, cR1–cR3, and cDe1–cDe3 represent the slopes of daytime dysfunction, social rejection, and depressive symptoms from T1 to T3 at the within‐individual level. RI—Da, RI—R, and RI—De represent the random intercepts of daytime dysfunction, social rejection, and depressive symptoms, respectively. All variables at each time point represent observed composite scale scores rather than latent constructs; item parceling was not applied.

First, an RI‐CLPM was established to investigate the mediating role of social rejection between daytime dysfunction and depressive symptoms. The modeled relationships among variables are depicted in Figure 5. The model showed a good fit: *χ^2^/df* = 6.333, RMSEA [90% CI] = 0.034 [0.000, 0.072], CFI = 0.998, TLI = 0.979, SRMR = 0.065.

At the between‐individual level, the association between daytime dysfunction and social rejection was not significant *(β* = −0.112, SE = 0.083*, p* = 0.178). Similarly, the relationship between social rejection and depressive symptoms was statistically significant *(β* = 0.290, SE = 0.079, *p <* 0.001), suggesting that adolescents who experienced greater social rejection tended to report more severe depressive symptoms. However, a significant positive association was found between daytime dysfunction and depressive symptoms *(β* = 0.633, SE = 0.051, *p* < 0.001), indicating that higher levels of daytime dysfunction were associated with more severe depressive symptoms in adolescents.

At the within‐individual level, from T2 to T3, social rejection and depressive symptoms significantly predicted their respective levels. Additionally, social rejection at T1 significantly predicted daytime dysfunction at T2. In contrast, the autoregressive paths of daytime dysfunction were not significant at any time point, and the autoregressive coefficients for social rejection and depressive symptoms from T1 to T2 were not significant. Furthermore, other cross‐lagged paths did not reach statistical significance. No significant mediating effects were observed in the model.

**FIGURE 6:**
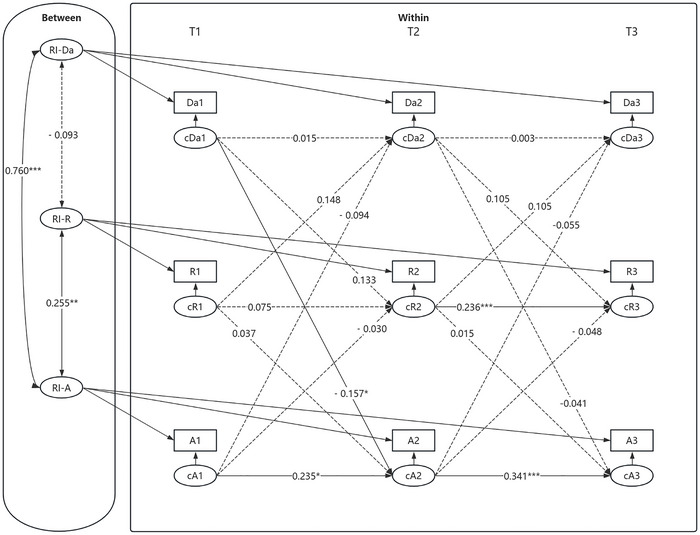
RI‐CLPM of daytime dysfunction, social rejection, and anxiety symptoms. *p < 0.05, **p < 0.01, ***p < 0.001. Da1–Da3, R1–R3, and A1–A3 represent the observed values of daytime dysfunction, social rejection, and anxiety symptoms at T1 to T3, respectively. cDa1–cDa3, cR1–cR3, and cA1–cA3 denote the individual—level slopes of daytime dysfunction, social rejection, and anxiety symptoms from T1 to T3. RI—Da, RI—R, and RI—A represent the random intercepts of daytime dysfunction, social rejection, and anxiety symptoms, respectively. All variables at each time point represent observed composite scale scores rather than latent constructs; item parceling was not applied.

Second, an RI‐CLPM was established to investigate the mediating role of social rejection between daytime dysfunction and anxiety symptoms. The modeled relationships among variables are depicted in Figure 6. Model fit indices indicated a good fit: *χ^2^/df* = 5.379, RMSEA [90% CI] = 0.029 [0.000, 0.068], CFI = 0.999, TLI = 0.985, SRMR = 0.015.

At the between‐individual level, the association between daytime dysfunction and social rejection was not significant *(β* = −0.093, SE = 0.081, *p* = 0.249). However, social rejection was positively associated with anxiety symptoms *(β* = 0.255, SE = 0.097, *p* < 0.01), suggesting that adolescents who experienced greater social rejection tended to report more severe anxiety symptoms. Additionally, daytime dysfunction was significantly and positively related to anxiety symptoms *(β* = 0.760, SE = 0.074, *p* < 0.001), indicating that adolescents with higher levels of daytime dysfunction were more likely to experience elevated anxiety symptoms.

At the within‐individual level, social rejection at T2 significantly predicted social rejection at T3, and anxiety at T(n) significantly predicted anxiety symptoms at T(n+1). Additionally, daytime dysfunction at T1 significantly predicted anxiety symptoms at T2. In contrast, the autoregressive paths for daytime dysfunction across all three time points were non‐significant. Social rejection at T1 did not significantly predict social rejection at T2. Furthermore, other cross‐lagged paths did not reach statistical significance. Mediation analysis revealed no significant indirect effects through social rejection.

## 4 Discussion

Using three‐wave longitudinal data from Chinese adolescents, we fitted RI‐CLPMs to test direct and indirect pathways linking daytime dysfunction to depressive and anxiety symptoms. Results showed bidirectional effects between daytime dysfunction and both depressive and anxiety symptoms, with social neglect partially mediating the impact of daytime dysfunction on subsequent anxiety symptoms.

### 4.1 The Relationship between Daytime Dysfunction, Depressive, and Anxiety Symptoms

At the between‐person level, the random intercepts of daytime dysfunction, depressive symptoms, and anxiety symptoms were positively correlated, indicating that adolescents with chronically elevated daytime dysfunction also exhibited more severe trait‐like depressive and anxiety symptoms (Liu et al. 2018). This association likely reflects the detrimental impact of persistent daytime dysfunction on neurocognitive processes—namely, diminished emotion‐regulation capacity, heightened negative affect, and attenuated stress coping—which together increase vulnerability to internalizing problems (Curtis et al. 2018; Xu et al. 2023).

After partialing out these stable, trait‐like components, significant reciprocal within‐person effects remained. Fluctuations in daytime dysfunction predicted subsequent increases in both depressive and anxiety symptoms, and vice versa, corroborating prior evidence for the short‐ and long‐term emotional sequelae of daytime dysfunction (Shim et al. 2019; Spira et al. 2008). The present three‐wave study extends these findings by demonstrating that transient deteriorations in daytime functioning exert sustained, cross‐lagged effects on emotional distress, thereby underscoring daytime dysfunction as a critical risk factor for adolescent depressive and anxiety symptoms (Yin et al. 2023).

### 4.2 The Mediating Role of Social Ostracism

To delineate the mechanisms linking daytime dysfunction to adolescent emotional distress, we differentiated social ostracism into social neglect and social rejection and tested their longitudinal mediating effects within four RI‐CLPMs. At the within‐person level, only social neglect mediated the effect of daytime dysfunction on anxiety symptoms: T1 daytime dysfunction predicted increased T2 social neglect, which in turn predicted higher T3 anxiety symptoms. This cross‐time sequence indicates that subtle, cumulative neglect translates transient sleep‐related impairments into sustained anxiety symptoms (Jiang et al. 2019). By contrast, social neglect did not mediate the daytime dysfunction–depressive symptoms relation, consistent with evidence that depressive symptoms are more strongly influenced by enduring cognitive–affective processes than by immediate social fluctuations (Monroe et al. 2007). The unobtrusive nature of neglect may further deplete self‐regulatory resources, thereby heightening anxiety symptoms (Williams 2009).

On the other hand, social rejection did not show a significant mediating role. Although rejection exhibited temporal stability, it did not transmit the influence of daytime dysfunction to depressive or anxiety symptoms. The salience of overt rejection may prompt rapid coping responses—such as support seeking or self‐affirmation—that attenuate its emotional impact (Xie et al. 2023). This fits with emotion regulation theory, which suggests people use different strategies to manage emotions and adapt to changes (Gross 2002).

At the between‐person level, the association between social rejection and more severe depressive and anxiety symptoms may be explained by several factors. First, rejected individuals tend to be more sensitive to negative social cues and are prone to cognitive biases, such as overinterpreting others' behavior as hostility (Peng et al. 2023), which could intensify depressive and anxiety symptoms. Additionally, social rejection undermines individuals' self‐esteem (Jenchura et al. 2017) and reduces self‐efficacy, making them feel more helpless and anxious when facing social challenges (Thyagaraj et al. 2025). Furthermore, the lack of social support makes it harder for individuals to regulate their emotions in stressful situations, leading to accumulated depressive and anxiety symptoms (Miller et al. 2024).

In sum, social neglect, as a covert form of ostracism, uniquely mediates the longitudinal pathway from daytime dysfunction to adolescent anxiety symptoms, whereas social rejection does not serve a comparable mediating role. Preventive and clinical efforts should therefore prioritize the identification and remediation of chronic social neglect in adolescents’ daily environments.

### 4.3 Limitations of the Study

Several limitations must be acknowledged. First, T1 data were collected during summer vacation, potentially affecting baseline measurements. Second, reliance on self‐report introduces potential biases. Future studies should incorporate behavioral or physiological indicators (such as HRV and EEG) for triangulation. Third, the sample size, while adequate, warrants replication in larger cohorts. Fourth, the longitudinal stability coefficients for all constructs are notably low. This limits the interpretability of the cross‐lagged effects. Future studies using longer follow‐up intervals, additional assessment waves, or alternative measurement approaches are needed to more robustly capture developmental stability and change. Finally, daytime dysfunction was treated as a single dimension; future research could differentiate sleep subtypes (such as insomnia onset and maintenance) to refine the model.

## 5 Conclusion

Three‐wave longitudinal data demonstrated that adolescent daytime dysfunction prospectively predicts both depressive and anxiety symptoms, confirming its status as a salient risk factor for emotional maladjustment. Further analysis indicated that social neglect longitudinally mediates the effect of daytime dysfunction on anxiety symptoms. These findings underscore the need to incorporate assessments of daytime dysfunction and social exclusion into adolescent psychological interventions and offer a novel psychosocial framework for understanding sleep‐related emotional symptoms.

## Author Contributions

W.W.J. conceived of the study, supervised the project, and participated in the revision of the manuscript. D.D.Y. contributed to the supervision, interpretation of the findings, and critical revision of the manuscript. Z.N. led the drafting of the original manuscript and contributed to reviewing and editing. J.C.G. conducted data analysis and contributed to manuscript review. T.S.Y. participated in manuscript writing and revision. L.T.C. was responsible for data curation and visualization. W.C.C., H.Y.H., L.J.Z., Z.Z.Y., and F.D.Y. were involved in investigation and formal analysis. All authors read and approved the final manuscript.

## Funding

This work was supported by the Anhui Provincial Department of Education (Anhui Province Scientific Research Planning Key Project (Grant No. 2022AH051512), the Anhui Province Postgraduate Quality Engineering Project (Grant No. 2024zyxwjxalk187), the Anhui Province Postgraduate Innovation and Entrepreneurship Practice Project(Grant No. 2023cxcysj160), and the Innovative Training Program for Chinese College Students(Grant Nos. bydc2024072, bydc2024073, bydc2024078).

## Conflicts of Interest

The authors declare no competing interests.

## Data Availability

The datasets used or analyzed during the current study are available from the corresponding author upon reasonable request.
